# Early outcome of early-goal directed therapy for patients with sepsis or septic shock: a systematic review and meta-analysis of randomized controlled trials

**DOI:** 10.18632/oncotarget.15550

**Published:** 2017-02-20

**Authors:** Xiaofan Chen, Weifeng Zhu, Jing Tan, Heyun Nie, Liangming Liu, Dongmei Yan, Xu Zhou, Xin Sun

**Affiliations:** ^1^ Chinese Cochrane Centre, Chinese Evidence-Based Medicine Centre, West China Hospital, Sichuan University, Chengdu, Sichuang, China; ^2^ Evidence-Based Medicine Research Centre, Jiangxi University of Traditional Chinese Medicine, Nanchang, Jiangxi, China; ^3^ State Key Laboratory of Trauma, Burns and Combined Injury, Second Department of Research Institute of Surgery, Daping Hospital, Third Military Medical University, Chongqing, China

**Keywords:** early-goal directed therapy, sepsis, septic shock, randomized controlled trial, meta-analysis

## Abstract

Various trials and meta-analyses have reported conflicting results concerning the application of early goal-directed therapy (EGDT) for sepsis and septic shock. The aim of this study was to update the evidence by performing a systematic review and meta-analysis. Multiple databases were searched from initial through August, 2016 for randomized controlled trials (RCTs) which investigated the associations between the use of EGDT and mortality in patients with sepsis or septic shock. Meta-analysis was performed using random-effects model and heterogeneity was examined through subgroup analyses. The primary outcome of interest was patient all-cause mortality including hospital or ICU mortality. Seventeen RCTs including 6207 participants with 3234 in the EGDT group and 2973 in the control group were eligible for this study. Meta-analysis showed that EGDT did not significantly reduce hospital or intensive care unit (ICU) mortality (relative risk [RR] 0.89, 95% CI 0.78 to 1.02) compared with control group for patients with sepsis or septic shock. The findings of subgroup analyses stratified by study region, number of research center, year of enrollment, clinical setting, sample size, timing of EGDT almost remained constant with that of the primary analysis. Our findings provide evidence that EGDT offers neutral survival effects for patients with sepsis or septic shock. Further meta-analyses based on larger well-designed RCTs or individual patient data meta-analysis are required to explore the survival benefits of EDGT in patients with sepsis or septic shock.

## INTRODUCTION

Severe sepsis and septic shock are one of the commonest life-threatening conditions in critically ill patients with a high mortality rate ranging from 15% to 50% [1, 2]. First reported in 2001 by Rivers et al in a randomized controlled trial (RCT), the mortality rate of severe sepsis and septic shock reduced to a lower level if a specific six-hour resuscitation bundle of early-goal directed therapy (EGDT) was given [3].

Though since 2004, Surviving Sepsis Campaign (SSC) Guidelines has been advocated and updated every four years, it remained to be a big challenge for such patients [4–6]. In the past decade, more and more large-scale RCTs and observational studies have been conducted with controversial results for EGDT [7–17]. Some studies reported survival benefits [10–13, 17] while others, especially some recent RCTs and meta-analysis showed no survival benefits for patients receiving EGDT compared with those with usual care [7–9, 18–20]. We performed this systematic review aimed at updating the current evidence from RCTs to determine the survival effect of EGDT compared with that of usual care in sepsis and septic shock patients.

## RESULTS

### Description of the included trials

**Figure 1:**
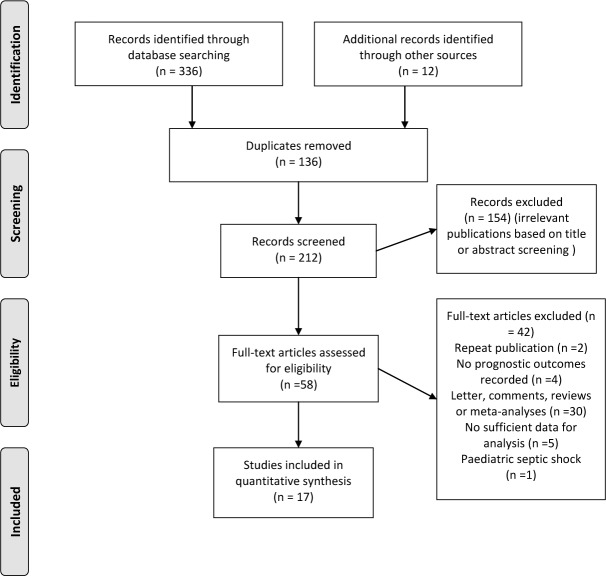
Flow diagram of literature search for trials investigating association between early-goal directed therapy and hospital/ICU mortality for sepsis and septic shock

The initial literature search yielded a total of 348 references for eligibility. After excluding the duplicates and further screening the titles or abstracts, 17 trials were identified that met our inclusion criteria (Figure 1) [7–9, 11–17, 21–27].

We presented the baseline characteristics of each included trial concerning study design, participant features, interventions, and study characteristics in Table 1. In summary, 17 RCTs including 6207 participants with 3234 in the experimental group and 2973 in the control group were enrolled for analysis. The study sample size ranged from 33 to 1600 participants. Seven trials were conducted in USA, 5 in Europe and 5 in Asia. Eight trials recruted participants in multiple centres, while 9 in single centre. As for clinical setting, 11 trials included participants in intensive care units, 4 in emergency department and 2 in both settings. The timing of EGDT were 6-10 hours (hrs) in 11 trials and≥24 hrs in 2 trials.

**Table 1:** Summary characteristics of randomised controlled trials included in systematic review and meta-analysis on early-goal directed therapy and hospital/ICU mortality for sepsis and septic shock

Trial	Year	Country	Single/multiple center	Initiation of enrollment	No. of patients (EGDT/control)	Study population	Clinical setting	Goals in EGDT group	Goals in control group	Timing of EGDT	Endpoint
Tuchschmidt, et al.	1992	USA	Single	NR	26/25	Adult patients with septic shock	ICU	CI>6 L/min/m^2^SBP>90 mm Hg	CI>3 L/min/m^2^SBP>90 mm Hg	6 hrs	Hospital mortality
Yu, et al.	1993	USA	Multicenter	NR	30/22	Adult patients with septic shock	ICU	DO_2_I>600 mL/min/m^2^SBP>100 mm Hg	DO_2_I450–550 mL/min/m^2^	24 hrs	30 days mortality
Hayes, et al.	1994	UK	Multicenter	NR	50/50	Adult patients with septic shock	ICU	CI>4.5 L/min/m^2^DO_2_I>600 mL/min/m^2^VO_2_>170 mL/min/m^2^	Usual care	Unclear	Hospital mortality
Gattinoni, et al.	1995	Italy	Multicenter	1991	124/57	Adult patients with septic shock	ICU	CI>4.5 L/min/m^2^ or SvO_2_>70%MBP>65 mm HgCVP 8–12 mm HgUO>0.5 mL/kg/hr	CI 2.5–3.5 L/min/m^2^MBP>65 mm HgCVP 8–12 mm HgUO>0.5 mL/kg/hr	Unclear	180 days mortality
Alía, et al.	1999	Spain	Single	1993	31/32	Adult patients with septic shock	ICU	DO_2_I>600 mL/min/m^2^MBP>60 mm Hg	DO_2_I>330 mL/min/m^2^MBP>60 mm Hg	Unclear	ICU mortality
Rivers, et al.	2001	USA	Single	1997	130/133	Adult patients with septic shock	ED	SvO_2_>70%, CVP 8–12 mm Hg, MAP 65–90 mm Hg, UO>0.5 mL/kg/hr	CVP 8–12 mm HgMBP 65–90 mm HgUO>0.5 mL/kg/hr	6 hrs	Hospital mortality
Lin, et al.	2006	Taiwan	Single	2003	108/116	Adult patients with septic shock	ICU	CVP 8–12 mm Hg, MAP>65 mm Hg, UO>0.5 mL/kg/hr	Usual care	6 hrs	Hospital mortality
Wang, et al.	2006	China	Single	2004	16/17	Adult patients with septic shock	ICU	CVP 8–12 mm Hg, MAP>65 mm Hg, ScvO2>70%, UO>0.5 mL/kg/hr	Usual care	6-10 hrs	Hospital mortality (7 days and 14 days)
Jones, et al.	2010	USA	Multicenter	2007	150/150	Adult patients with septic shock	ED	ScvO_2_>70%, CVP 8–12 mm Hg, MAP 65–90 mm Hg, UO>0.5 mL/kg/hr	Lactate clearanceCVP>8 mm HgMBP>65 mm Hg	Unclear	Hospital mortality
The EDGT Collaborative Group of Zhejiang Province	2010	China	Multicenter	2005	163/151	Adult patients with severe sepsis and septic shock	ICU	CVP 8–12 mm Hg, MAP>65 mm Hg, SBP>90mmHg, UO>0.5 mL/kg/hr, ScvO2>70%	CVP 8–12 mm Hg, MAP>65 mm Hg, SBP>90mmHg, UO>0.5 mL/kg/hr	6 hrs	Hospital mortality (28 days)
Tian, et al.	2012	China	Single	2009	43/19	Adult patients with septic shock	ICU	CVP 8–12 mm Hg, MAP>65 mm Hg, ScvO2>70%, UO>0.5 mL/kg/hr, 6 h LCR>10% or 30%	CVP 8–12 mm Hg, MAP>65 mm Hg, ScvO2>70%, UO>0.5 mL/kg/hr	6 hrs	Hospital mortality (7 days and 28 days)
Yu, et al.	2013	China	Single	2011	25/25	Adult patients with severe sepsis and septic shock	ICU	CVP≥8mmHg, MAP≥65mmHg, LCR≥10%	CVP≥8mmHg, MAP≥65mmHg, ScvO2≥0.70	6 hrs	Hospital mortality (28 days)
Andrews, et al.	2014	USA	Single	2012	53/56	Adult patients with septic shock	EDward ICU	Simplified Severe Sepsis Protocol: Hb>7 initial 2 L bolus of NS (within 1 hr), if, CVP<3 mm Hg; 2 L loading MAP>65 mm Hg, dopamine infusion 10 mcg/kg/min	ScvO2≥0.70	6 hrs	Hospital mortality
ARiSe	2014	Australia or New Zealand	Multicenter	2008	796/804	Adult patients with septic shock	ED	ScvO_2_>70%, CVP 8–12 mm Hg, MAP 65–90 mm Hg, UO>0.5 mL/kg/hr	Usual care	6 hrs	90 days mortality
ProCESS	2014	USA	Multicenter	2008	439/456	Adult patients with septic shock	ED	ScvO_2_>70%, CVP 8–12 mm Hg, MAP 65–90 mm Hg, UO>0.5 mL/kg/hr	Usual care	6 hrs	60 days mortality
Lu, et al.	2014	China	Single	2009	42/40	Adult patients with septic shock	ICU	ITBVI 850-1500 mL/m^2^, MAP≥65 mmHg	CVP 8–12 mm Hg, MAP>65 mm Hg, ScvO2>70%, UO>0.5 mL/kg/hr	72 hrs	Hospital mortality
ProMISe	2015	UK	Multicenter	2012	623/620	Adult patients with septic shock	EDICU	ScvO_2_>70%, CVP 8–12 mm Hg, MAP 65–90 mm Hg, UO>0.5 mL/kg/hr	Usual care	6 hrs	90 days mortality

Abbreviations: CI, cardiac output; CVP central venous pressure; ED emergency department; DO_2_I, oxygen delivery index; EGDT, early-goal directed therapy; hr, hour; ICU, intensive care unit; LCR, lactate clearance rate; ITBVI, intrathoracic blood volume index; MAP, mean arterial pressure; ScvO2, central venous oxygen saturation; UO, urine output.

### Early-goal directed therapy and hospital/ICU mortality

**Figure 2:**
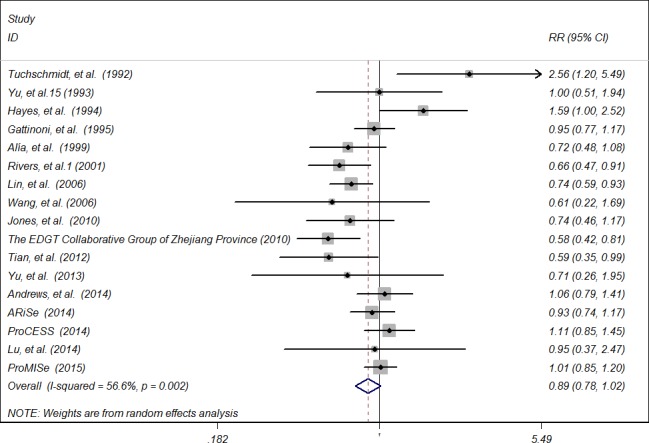
Forest plot of randomised controlled trials of early-goal directed therapy and hospital/ICU mortality for sepsis and septic shock Weights from random effects analysis.

A meta-analysis of 17 RCTs including 6207 participants, showed that early-goal directed therapy did not significantly reduce hospital/ICU mortality (relative risk [RR] 0.89, 95% CI 0.78 to 1.02) compared with control group for patients with sepsis or septic shock (Figure 2), with significantly heterogeneous (I^2^ = 56.6%) among trials. The findings of subgroup analyses stratified by trial region, number of research center, year of enrollment, clinical setting, sample size, timing of EGDT almost remained constant with that of the main analysis, except that for trials conducted in Asian coutries, patients treated with early-goal directed therapy had significant reduced hospital/ICU mortality (RR 0.68, 95% CI 0.57 to 0.80) compared with those in control group. A bordine effect was noted for trials with sample size more than 100 (RR 0.87, 95% CI 0.76 to 1.00).

### Trial center

Nine and eight trials were conducted in single center and mutiple centers, respectively. The summary RRs estimated for hospital or ICU mortality were 0.82 (95% CI 0.65 to 1.02) for single center and 0.95 (95% CI 0.81 to 1.11 ) for mutiple centers. Statistically significant difference for inter-study heterogeneity (*P* = 0.005) was noted.

### Year of enrollment

Three trials were performed before year 2000, with the summary RRs for hospital or ICU mortality of 0.79 (95% CI 0.62 to 1.02). Eight and three trials were performed between year 2000-2010 and after year 2010, with the summary RRs for hospital or ICU mortality of 0.79 (95% CI 0.67 to 0.97) and 1.07 (95% CI 0.88 to 1.30), respectively. There was no significant difference for inter-study heterogeneity (*P* = 0.055).

### Sample size

The pooled RRs for hospital or ICU mortality stratified by sample size were 0.87 (95% CI 0.76 to 1.00) for trials with large sample size (<100) and 0.97 (95% CI 0.67 to 1.40) for trials with small sample size (≤100). We found statistically significant difference for inter-study heterogeneity (*P* = 0.923).

### Trial region

Seven trials were conducted in USA, and another 7 and 5 studies were in Europe and Asia, respectively. The pooled RRs for hospital or ICU mortality were 0.98 (95% CI 0.76 to 1.27) for trials conducted in USA, 0.98 (95% CI 0.84 to 1.14) in Europe and 0.68 (95% CI 0.57 to 0.80) in Asia. Statistically significant difference for inter-study heterogeneity (*P* = 0.004) was noted.

### Inclusion period

Eleven trials enrolled patients in intensive care unit, with a pooled RR of 0.87 (95% CI 0.69 to 1.08); 4 included patients in emergency department with the pooled RR of 0.87 (95% CI 0.69 to 1.09) and two trials enrolled patients in the combined units of both intensive care unit and emergency department, with the summary RR of 1.02 (95% CI 0.88 to 1.19). We found statistically significant difference for inter-study heterogeneity (*P* = 0.037).

### Timing of EGDT

The summarised RRs for hospital or ICU mortality stratified by timing of EGDT were 0.87 (95% CI 0.73 to 1.03) for trials with patients having EGDT in 6-10 hours and 0.98 (95% CI 0.57 to 1.69) for trials with patients having EGDT after 24 hours. There was no statistically significant difference for heterogeneity among trials (*P* = 0.40).

### Publication bias

**Figure 3:**
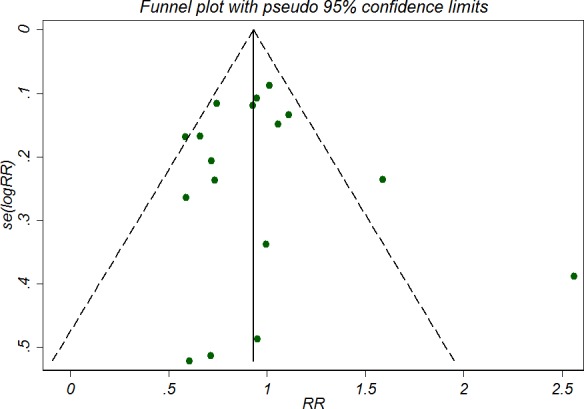
Funnel plot for the outcome of hospital/ICU mortality The tests for funnel plot asymmetry by Egger's test identified no publication bias (Egger's test, *P* = 0.74).

There was no significant funnel plot asymmetry as was shown in Figure 3. Symmetrical distribution of the trials on the funnel plot indicates no publication bias. Egger's regression intercept was 0.33 (*P* = 0.74) further suggesting no publication bias. Sensitivity analysis using Duval and Tweedie's trim and fill method suggested that no missing trial was inputed and the adjusted estimate was the same as the primary analysis, confirming the robustness of the analysis.

## DISCUSSION

This systematic review and meta-analysis involving the available RCTs on the impact of EGDT on hospital or ICU mortality for patients with sepsis and septic shock did not show a significant reduced risk of hospital/ICU all-cause mortality associated with the use of EGDT. Meanwhile, the findings were independent of trial region, number of research center, year of enrollment, clinical setting, sample size or timing of EGDT.

Several factors contribute to the development of sepsis or septic shock. Inflammatory mediators may lead to microcirculatory disturbance [28], and subsequent reduced perfusion and hypoxia could occur [29]. The primary goal of fluid resuscitation in sepsis or septic shock is to preserve effective circulatory blood volume, restore sufficient tissue perfusion, and sustain a balanced body oxygen uptake. The commonly used indicators in clinics for patients with sepsis or septic shock include mean arterial pressure (MAP), central venous pressure (CVP) and superior vena cava oxygenation saturation (ScvO2)/ mixed venous oxygen saturation (SvO2), which are suggested indicators for maintain the balance of volume homeostasis based on the EGDT criteria. The perfusion condition of tissue or organs, mainly indicated by CVP or MAP, could guide physicians’ decisions on the adjustment for the dosage of vasoactive agents accordingly. Besides, ScvO2 being more than 70% was also suggested a good indicator for the balance of supply and consumption of oxygen. It has been reported that the balance of oxygen supply plays a pivotal role for the mortality of patients with sepsis or septic shock. Moreover, several clinical trials provided evidence that implementing EGDT was significantly associated with reduced mortality in patients with sepsis or septic shock compared with those using conventional therapy [11–13, 17].

This meta-analysis combined evidence from 17 RCTs and focused on whether the application of EGDT with the indicators such as CVP, MAP, ScvO_2_ or SvO_2_ and Urine Output (UO) has the potential to reduce short-term mortality in patients with sepsis or septic shock. To the best of our knowledge, this study has the largest sample size of over 6200 participants compared with those published in recent years and obtain consistent neutral results with several meta-analyses.

We acknowledge that this meta-analysis should be interpreted with multiple limitations. Firstly, due to the broaden of our inclusion criteria with a total of 17 RCTs finally included, the heterogeneity of the pooled estimate was predictably significant (I^2^ = 56.2%) for the feature of trial population (patient age, disease severity and baseline health status), intervention treatment in trial group and in comparisons (difference in goals of EDGT protocol), outcome measurement (6h mortality, 24h mortality, etc.) and methodology quality (differenc in study design of RCTs), which we had investigated in subgroup analyses as presented in Table 2. Though the finding provided neither benefits nor harm of EGDT, we still could not conclude that EGDT was of no use for sepsis or septic shock, at least it was no bad than usual care. Secondly, we chose 6h or 24h mortality as study endpoints, which may have a potential impact on the combined estimates. However, we conducted subgroup analysis based on different mortality time interval with consistent findings. Thirdly, due to the unavailablitly of detailed information of each trial, some baseline features about patients conditions could not be abstracted to conduct sensitivity analysis. Moreover, though subgroup analyses had been conducted, still some of the causes of heterogeneity could not be found. We proposed that other potential sources of heterogeneity could lie in the characteristics of the enrolled populations such as the baseline disease status and treatment protocol.

**Table 2:** Subgroup analyses for relative risk of hospital/ICU mortality for patients with sepsis and septic shock receiving early-goal directed therapy compared with those with usual care

Comparison variables	Hospital/ICU mortality		
	(I^2^ statistics %; *P*_het_)	RR 95% CI	*P*_interaction_
Total	17(56.6; 0.002)	0.89 (0.78 to 1.02)	NA
Trial region			0.004
USA	7 (58.1, 0.026)	0.98 (0.76 to 1.27)	
Europe	5 (41.8, 0.143)	0.98 (0.84 to 1.14)	
Asia	5 (0, 0.775)	0.68 (0.57 to 0.80)	
Research center			0.005
Single-centered	9 (51.0, 0.038)	0.82 (0.65 to 1.02)	
Multiple-centered	8 (56.4, 0.025)	0.95 (0.81 to 1.11)	
Year of enrollment			0.066
∼2000	3 (49.3, 0.139)	0.79 (0.62 to 1.02)	
2000-2010	8 (48.4, 0.059)	0.79 (0.67 to 0.97)	
2010∼	3 (0, 0.701)	1.07 (0.88 to 1.30)	
Clinical setting			0.037
Intensive care unit	11 (61.3, 0.004)	0.87 (0.69 to 1.08)	
Emergency department	4 (55.5, 0.080)	0.87 (0.69 to 1.09)	
Combined	2 (0, 0.796)	1.02 (0.88 to 1.19)	
Sample size			0.923
≤100	8 (60.6, 0.013)	0.97 (0.67 to 1.40)	
>100	9 (58.2, 0.014)	0.87 (0.76 to 1.00)	
Timing of EGDT			0.40
6-10 hrs	11 (65.3, 0.001)	0.87 (0.73 to 1.03)	
≥24 hrs	2 (0, 0.937)	0.98 (0.57 to 1.69)	

Abbreviations: CI, confidence interval; EGDT, early-goal directed therapy; het, heterogeneity; hrs, hours;ICU, intensive care unit; RR, relative risk; NA, not available.

The present analysis has several strengths. Firstly, we performed an exhaustive literature search to identify all potential relevant trials from the main databases and the original authors of some of the trials were contacted *via* email and to have some additional data for analyses if possible, which have minimized the potential risk of publication bias. Though unpublished grey literature were not searched for insufficient data, the included trials cover countries from all over the European, American and Asian counties. Secondly, compared with meta-analysis of observational studies, the current study included only RCTs, providing direct evidence for the effect of EGDT on survival of patients with sepsis or septic shock. Thirdly, to explore the robustness of the findings, we applied sensitivity analysis through trim and filled method, and the consistent finding with the primary one was indicated. We proposed that for future trial design, larger number of patients with the same disease stage or severity should be enrolled to increase the statistical power.

In summary, current evidence from RCTs shows that EGDT adds no survival benefit or harm to patients with sepsis or septic shock. Further meta-analyses based on high-level RCTs or individual patient data meta-analysis are required to explore the survival benefits of EDGT in patients with sepsis or septic shock.

## MATERIALS AND METHODS

### Literature search and study selection

We conducted this systematic literature review according to the Preferred reporting items for systematic reviews and meta-analyses (PRISMA) statement [30]. A literature search of Pubmed, EMBASE and the Cochrane Library Central Register of Controlled Trials databases were searched from inception till August, 2016 for relevant citations, using search strategies (supplementary Search Strategy) in combination with exploded MeSH terms and text words concerning sepsis/septicaemia/septic shock, goal directed therapy/goal directed resuscitation/EGDT/GDT. Manual reference search from the primary selected reference lists was also performed for additional potential publications.

Trials were considered eligible for inclusion if they satisfied the following criteria elements (Participants, Intervention, Comparison, Outcome, Study design):

(1) Participants: Adult patients with severe sepsis and septic shock, treating with EGDT methods;

(2) Intervention: We used the standard EGDT, which was defined as a protocol resuscitation in accord with achieving specific therapeutic results in terms of CVP (8-12mmHg), MAP (65-90mmHg), UO (≥0.5ml/kg/h), and continuous monitoring to keep ScvO2 (≥70%).

(3) Comparison: Usual care or other protocols were adopted in the control group based on the included trials.

(4) Outcome: We set the primary outcome measure as all-cause mortality at the early period for patient treatment during hospitalization (hospital or ICU mortality). All-cause mortality was defined as the time from the trial randomization to death from any cause.

(5) Trial design: RCTs published with full texts and in peer-review journals without language restrictions. We excluded those non-RCTs or conference abstracts due to the high risk of bias.

### Data extraction and bias assessment

Two investigators (X.C. and W.Z.) independently screened, identified the citations and extracted baseline characteristics and evaluated the bias of each study from the selected trials. Any discrepancies were resolved through discussion with a third senior investigator (X.S or L.L.) until a consensus was reached. The characteristics abstracted were trial author, publication year, research country, number of trial center, initiation of enrollment, number of patients in trial group and control group, trial population, clinical setting, goals in trial group and in control group, timing of EGDT and trial endpoints.

The Cochrane Collaboration risk of bias tool was used to assess methodological quality of each trial according to the seven domains including adequacy of sequence generation, allocation concealment, blinding of participants, blinding for outcome assessment, incomplete outcome data, selective outcome reporting and other potential sources of bias [31].

### Statistical analysis

We conducted meta-analysis using the software STATA version 12.0 (StataCorp LP, College Station, TX). We set hospital mortality or ICU mortality as the primary outcome measure. For dichotomous data, we calculated the RR with 95% CI to assess the treatment effect and pooled using random-effects model [32] due to the proposed high between-trial variation. Inter-trial heterogeneity was examined using I^2^ statistics with an I^2^ value of more than 50% indicating significant heterogeneity [33]. Between-study heterogeneity were examined using subgroup analyses by stratifying study baseline characteristics such as trial region, number of research center, year of enrollment, clinical setting, sample size, timing of EGDT. Publication bias was tested by visual inspection of funnel plot asymmetry combined with Egger's test for statistical significance [34]. Duval and Tweedie's trim and fill method was also applied to further assess the robustness of the summary estimates [35].

## SUPPLEMENTARY MATERIALS TABLES


