# Supplementary material for: Early outcome of early-goal directed therapy for patients with sepsis or septic shock: a systematic review and meta-analysis of randomized controlled trials
Source: Oncotarget. 2017 Feb 20;8(16):27510–9. doi: 10.18632/oncotarget.15550 (PMC5432353; doi:10.18632/oncotarget.15550)
Supplement: Supplementary file 1 [file oncotarget-08-27510-s001.pdf]

Early outcome of early-goal directed therapy for patients with sepsis or septic shock: a systematic review and meta-analysis of randomized controlled trials

Supplementary Material

Search Strategy

Search included: Pubmed, Embase, and the Cochrane Library Central Register of Controlled Trials: till August, 2016

Table A1. Pubmed Search strategy

1. " Sepsis "[Mesh]
2. " Shock, Septic "[Mesh]
3. 1 OR 2
4. (sepsis OR septicaemia OR septic shock) [Title/Abstract]
5. 3 OR 4
6. " goal directed therapy "[Title/Abstract]
7. "goal directed resuscitation"[Title/Abstract]
8. "EGDT"[Title/Abstract]
9. "GDT"[Title/Abstract]
10. 6 OR 7 OR 8 OR 9
11. 5 OR 10
12. (randomized controlled trial OR controlled clinical trial OR randomized OR randomised OR placebo clinical trial OR randomly OR trial) [Title/Abstract]
13. 5 AND 11 AND 12

Table A2. Embase Search strategy

1. 'sepsis'/exp
2. 'septic shock'/exp
3. 1 OR 2
4. (sepsis OR septicaemia OR septic shock):ab,ti
5. 3 OR 4
6. ('goal directed therapy' OR 'goal directed resuscitation' OR EGDT OR GDT):ab,ti
7. (randomized controlled trial OR controlled clinical trial OR randomized OR randomised OR placebo clinical trial OR randomly OR trial) :ab,ti
8. 5 AND 6 AND 6

Table A3. Search strategy for the Cochrane Library Central Register of Controlled Trials

1.	MeSH descriptor: [Sepsis] explode all trees
2.	MeSH descriptor: [Shock, Septic] explode all trees
3.	1 OR 2
4.	(sepsis or septicaemia or septic shock):ti,ab,kw
5.	3 OR 4
6.	(‘goal directed therapy’ or ‘goal directed resuscitation’ or EGDT or GDT):ti,ab,kw (Word variations have been searched)
7.	(randomized controlled trial or controlled clinical trial or randomized or randomised or placebo clinical trial or randomly or trial):ti,ab,kw (Word variations have been searched)
8.	5 AND 6 AND 7
